# Evaluation of Agreement between HRT III and iVue OCT in Glaucoma and Ocular Hypertension Patients

**DOI:** 10.1155/2015/691031

**Published:** 2015-12-15

**Authors:** A. Perdicchi, M. Iester, D. Iacovello, A. Cutini, M. Balestrieri, M. G. Mutolo, A. Ferreras, M. T. Contestabile, S. M. Recupero

**Affiliations:** ^1^Department of Ophthalmology II, Faculty of Medicine and Psychology, “Sapienza” University of Rome, Sant'Andrea Hospital, 00100 Rome, Italy; ^2^Anatomical-Clinical Laboratory for the Diagnosis and Treatment of Glaucoma and Neuroophthalmology, Eye Clinic, DINOGMI, University of Genoa, Viale Benedetto XV 5, 16132 Genoa, Italy; ^3^Department of Ophthalmology, Miguel Servet University Hospital, Aragon Health Research Institute, 50009 Zaragoza, Spain

## Abstract

*Purpose*. To determine the agreement between Moorfields Regression Analysis (MRA), Glaucoma Probability Score (GPS) of Heidelberg retinal tomograph (HRT III), and peripapillary nerve fibers thickness by iVue Optical Coherence Tomography (OCT).* Methods*. 72 eyes with ocular hypertension or primary open angle glaucoma (POAG) were included in the study: 54 eyes had normal visual fields (VF) and 18 had VF damage. All subjects performed achromatic 30° VF by Octopus Program G1X dynamic strategy and were imaged with HRT III and iVue OCT. Sectorial and global MRA, GPS, and OCT parameters were used for the analysis. Kappa statistic was used to assess the agreement between methods.* Results*. A significant agreement between iVue OCT and GPS for the inferotemporal quadrant (*κ*: 0.555) was found in patients with abnormal VF. A good overall agreement between GPS and MRA was found in all the eyes tested (*κ*: 0.511). A good agreement between iVue OCT and MRA was shown in the superonasal (*κ*: 0.656) and nasal (*κ*: 0.627) quadrants followed by the superotemporal (*κ*: 0.602) and inferotemporal (*κ*: 0.586) sectors in all the studied eyes.* Conclusion*. The highest percentages of agreement were found per quadrant of the MRA and the iVue OCT confirming that in glaucoma damage starts from the temporal hemiretina.

## 1. Introduction

Glaucoma is an optic neuropathy characterized by progressive damage of the retinal nerve fiber layer (RNFL) and the nerve head (ONH). It often precedes perimetric damage and in its most advanced stages leads to atrophy of the ONH [1–5]. Various diagnostic instruments have been used for evaluating RNFL thickness (RNFLt) and the morphometry of the ONH. Optic Coherence Tomography (OCT) and the Heidelberg Retina Tomograph (HRT; Heidelberg Engineering GMBH, Heidelberg, Germany) are the most used systems.

OCT is a high resolution imaging technique that permits direct measurement of retinal thickness [2] and monitoring treatment in retinal pathology. Numerous studies have shown a good correlation between RNFLt measured with this technique and that calculated on histological preparations [3].

The HRT is a confocal laser system that acquires three-dimensional topographic images of the ONH and the peripapillary area [4, 5]. Moorfields Regression Analysis (MRA) and Glaucoma Probability Score (GPS) are able to distinguish normal from glaucomatous eyes with a good diagnostic precision [6].

Numerous studies have been carried out for calculating the degree of correlation between RNFLt obtained with the first generation “Time Domain” OCT and ONH parameters calculated by HRT [7–11]. In recent years OCT has greatly developed with the introduction of the “spectral domain” technique for acquiring images. The spectral domain OCT system has a greater resolution power, allowing a more accurate analysis of anatomical structures which do not always agree with the information obtained with the time domain OCT [12–14]. One of the latest spectral domain OCT tools is the iVue OCT which has shown its value in the study of numerous retinal pathologies and in glaucoma.

The aim of this study was to assess agreement among HRT MRA and HRT GPS and RNFLt measurement with the iVue OCT in ocular hypertension (OH) and primary open angle glaucoma (POAG) patients.

## 2. Material and Methods

This was a prospectively cross-sectional study. The research followed the tenets of the Declaration of Helsinki and informed consent was obtained from all the patients included.

Patients were recruited from the clinics of one glaucoma specialist (AP), and they were not excluded on the basis of sex, age, or race. Seventy-two eyes of 40 patients were included in this study [15]. All the eyes were classified as OH or POAG. OH patients had a normal VF, ophthalmoscopically normal ONH, and an intraocular pressure (IOP) greater than 21 mmHg without therapy measured by i-Care tonometry [16], while POAG patients had abnormal VF with corresponding ONH damage by ophthalmoscopy and an IOP greater than 21 mmHg without therapy.

All patients had carried out a two-phase white on white dynamic strategy perimetry Octopus 1-2-3 Program G1X [17]. Mean Defect (MD) and Loss Variance (LV) values were taken to distinguish the visual fields between normal and pathological. After any phase all patients had performed IOP measurement to evaluate any IOP changes [18]. Visual fields with MD values between +2 dB and −2 dB and LV between 0 and 6 dB^2^ were considered normal and those with MD < −2 dB and LV > 6 dB^2^ were considered pathological. None of the patients had a MD > −10 dB and a LV > 10 dB^2^. The entire group was then divided into two subgroups based on the VF classification: normal VF subgroup and abnormal VF subgroup.

Each included eye underwent ONH analysis by HRT III and the RNFLt assessment by iVue OCT. The imaging examinations were performed on the same day, while the perimetric test was performed within 7 days.

### 2.1. iVue OCT

The ONH protocol of iVue OCT consists of 12 radial scans of 3.4 mm in length (452 A scans each) and 6 concentric ring scans ranging from 2.5 to 4.0 mm in diameter (587 to 775 A scans each), all centred on the optic disc. All the images were reprocessed with three-dimensional/video baseline. ONH parameters measured by the software included optic disc area, optic cup area, neuroretinal rim area, nerve head volume, cup volume, rim volume, cup-disc area ratio, horizontal cup-disc ratio, and vertical cup-disc ratio. The ONH protocol also generates a polar RNFL thickness map, measured along a circle 3.45 mm in diameter centred on the optic disc. It gives the average RNFLt in the temporal, superior, nasal, and inferior quadrants as well as the overall average along the entire measurement circle [19].

The software automatically calculates the disc margins, along the six radial scans, and uses an algorithm to automatically differentiate the microstructures of the retina that form part of the same RNFL [20, 21]. This automated computer algorithm separates the anterior and posterior margins of the identifying reflection group of the RNFL [22], making it possible to quantify thickness. These measurements can be compared with a database included in the iVue OCT.

#### 2.1.1. HRT

HRT is able to scan the retinal and optic nerve area surface at multiple consecutive parallel focal planes. These parameters have been shown to have a good sensitivity and specificity to detect glaucomatous ONH changes [23, 24].

However, there are 2 weak points in the HRT methodology: the reference plane and the contour line [25, 26] that are related to the capacity of the operator to detect the right size of the peripapillary area to be analyzed. The results obtained can be processed by several methods of analysis. One of the most used and significant is the MRA which has an extensive and specific database for various ethnic groups. It is formed of 948 eyes of which 733 belonged to Caucasians and 215 to blacks. In order to increase the diagnostic capacity of HRT, in 2000 Swindale et al. [27] published a new method to analyze ONHs without using a contour line by evaluating the shape of the ganglion cells when they cross the scleral canal. A good sensitivity and specificity was obtained and recently this new method called Glaucoma Probability Score (GPS) was applied to the HRT III [14].

For every single imaging test the values of the different quadrants were assessed; in particular the* inferotemporal* (IT),* inferonasal* (IN),* superotemporal* (ST),* superonasal* (SN),* nasal* (N), and* temporal* (T) ONH and RNFLt were considered. Furthermore, for MRA and GPS, a global (GLOBAL) index was calculated.

### 2.2. Statistical Analysis

In our study the percentages of concordance (n-n; b-b; a-a), relative concordance (n-b; b-a), and discordance (a-n) obtained with the different methods were calculated in relation to the VF damage classification (normal or abnormal VF). For the three devices six different sectors were considered, while for HRT MRA and GPS global indices were also considered.

Kappa statistic (*κ*) was used to study the agreement among the 3 different methods (MRA and GPS for HRT and OHN protocol for iVue) and between the methods. *κ* measures the change-corrected agreement on a scale of −1.0 to 1.0, with 1.0 indicating perfect agreement. We used the indications suggested by Landis and Koch: *κ*'s of 0.0 or less were considered to indicate poor; 0.0 to 0.2, slight; 0.21 to 0.4, fair; 0.41 to 0.6, moderate; 0.61 to 0.8, substantial; and 0.81 to 1, almost perfect agreement. In *κ* analysis only the agreement between normality and abnormality for the different methods was considered.

## 3. Results

On the basis of the results of VF examination, 72 eyes were recruited in the study and in particular 54 eyes had normal achromatic perimetry (MD between +2 and −2 dB and LV between 0 and 6 dB^2^) and 18 had visual field damage. Refractive error was −2.3 ± 3.2 diopters. Besides in the subgroup with normal VF the mean MD was 0.08 ± 0.98 dB and LV was 2.2 ± 0.93, while in the subgroup with abnormal VF the mean MD was −5.85 ± 5.82 dB and LV was 5.87 ± 2.39.

In the normal VF group the global index of HRT III classified 63% subjects as normal by using MRA and 28.5% by GPS, and borderline results were 24% and 28.5%, respectively, and abnormal results 11% and 41%, respectively, while in the glaucoma VF damage group, percentages were, respectively, 16.6% and 33.3% for normal, 27.7% and 33.3% for borderline, and 55.5% and 33.3% for abnormal.

Tables 1 and 2 show the percentage of concordance, relative concordance, and discordance for each single sector and globally (only HRT) among MRA, GPS, and iVue in patients with normal VF (Table 1) and abnormal VF (Table 2).

When iVue OCT and GPS data were compared, the *κ* statistic indicated in the eyes with abnormal VF a good agreement especially of the inferotemporal quadrant (*κ*: 0.555), while a low agreement was indicated for all the other sectors considered both in patients with normal and in patients with altered VF (Table 3).

When GPS and MRA data were compared, the *κ* statistic showed a good agreement in the global analysis (*κ*: 0.511). The agreement was more significant in the inferotemporal (*κ*: 0.618), inferonasal (*κ*: 0.527), and superonasal (*κ*: 0.519) quadrants. This result was confirmed also in the normal VF subgroup (inferotemporal *κ*: 0.516, inferonasal *κ*: 0.503, and superonasal *κ*: 0.508). A slightly different distribution was noted in patients with an abnormal VF where the global agreement between GPS and MRA was high (*κ*: 0.782) with greater significance that decreased from the inferotemporal (*κ*: 0.792), superotemporal (*κ*: 0.560), and inferonasal (*κ*: 0.526) to the superonasal (*κ*: 0.476) quadrant (Table 4).

When iVue and MRA data were compared, there was a good agreement in the superonasal (*κ*: 0.656) and nasal (*κ*: 0.627) quadrants followed by the superotemporal (*κ*: 0.602) and inferotemporal (*κ*: 0.585) sectors. When the normal VF subgroup was considered, it was found that the superonasal quadrant had the highest agreement (*κ*: 0.845) followed by the nasal quadrant (*κ*: 0.843), while when the abnormal VF subgroup was considered, a moderate significance was shown in the inferotemporal (*κ*: 0.545) and superotemporal (*κ*: 0.607) quadrants (Table 5).

## 4. Discussion

Glaucoma is an optic neuropathy characterized by specific and progressive ONH and RNFL damage. Consequently the ability to identify these alterations at the earliest possible stage is fundamental to start the treatment and to decrease the loss of ganglion cells which could carry to the atrophy of the ONH with functional loss and serious visual disability [1–4].

It is well known how time domain OCT appears to be highly sensitive and specific in identifying anatomic damage in the presence of manifest VF damage [8–11, 28] and these results were subsequently confirmed by studies done with spectral domain OCT [29–31].

The aim of our study was to determine if the parameters provided by a new instrument, a spectral domain OCT (iVue OCT), were in agreement, and to what extent, with those provided by the HRT III, more specifically with the GPS and with the MRA, in eyes with OH and/or glaucoma, both in the presence and in the absence of perimetric damage.

The comparison between iVue and GPS, two automatic methods of classification of the anatomical ONH and peripapillary RNFL damage, did not show a satisfactory agreement except for the inferotemporal (*κ*: 0.555) quadrant and only in eyes with an abnormal VF.

The comparison between GPS and MRA showed a good agreement in all the eyes examined, both those with normal VF and those with altered VF (*κ*: 0.511). In the abnormal VF subgroup this result increased (*κ*: 0.782) and the inferotemporal sector had the highest agreement (*κ*: 0.792). Similarly in the normal VF subgroup, the agreement between GPS and MRA was not significant when considered globally (*κ*: 0.391) but became significant when the inferotemporal (*κ*: 0.516) quadrant was considered. In the abnormal VF subgroup, better agreement was found between GPS and MRA outlining that GPS had similar sensitivity and specificity to MRA. This result is different from those of other studies in which MRA and linear regression analysis had higher ROC curves than GPS. In this study we did not evaluate the diagnostic capacity but only if the classification was similar and for this reason we probably obtained better results because no healthy normal subjects were included where usually GPS has a low specificity.

When the agreement between iVue OCT and MRA was considered, it was significant in eyes both with normal and with abnormal VF especially in the superonasal (*κ*: 0.656), nasal (*κ*: 0.627), and inferotemporal (*κ*: 0.585) quadrants. This tendency was confirmed in an analysis of eyes with both a normal and with an abnormal VF, even if in the latter the agreement was less significant.

Finally the agreement was good in the superonasal and nasal quadrant probably because these areas are the last to be involved in glaucoma, while the inferotemporal is the first to change.

In conclusion, the automatic methods of ONH analysis by GPS and peripapillary RNFL with the ONH protocol of the iVue OCT offer interesting application ideas but appear to have little agreement with one another. This weakness mainly regards eyes with a normal VF, where evidence of early anatomical damage is certainly of greatest interest. Moreover, our results suggest that the measurement of the RNFL using the HRT III and the iVue OCT is not interchangeable with the automatic methods (GPS), outlining that the computerized devices are able to analyze different anatomical structures. It has not yet been determined how useful and reliable these new objective techniques are for measuring anatomical damage when it comes to evaluating the progression of glaucoma over time. This is still the main goal to pursue in order to preserve good sight and consequently an acceptable quality of life for glaucoma patients.

## Figures and Tables

**Table 1:** (%) Normal VF.

	IT	IN	ST	SN	N	T	Global
GPS versus iVue							
Concordance	39	35.3	39	37.1	29	28.5	
Relative concordance	29.5	31.4	40.7	33.2	33.5	29	
Discordance	31.5	33.3	20.3	29.7	37.5	42.5	
MRA versus GPS							
Concordance	50	55	42.5	46	39.5	29.5	44.5
Relative concordance	37	26	39	39	40.5	46	39
Discordance	13	19	18.5	15	20	24.5	16.5
iVue versus MRA							
Concordance	61	50	64.5	63	55.5	74	
Relative concordance	26	31.5	26	24	24.5	24	
Discordance	13	18.5	9.5	13	20	2	

**Table 2:** (%) Glaucoma VF damage.

	IT	IN	ST	SN	N	T	Global
GPS versus iVue							
Concordance	55.3	33.4	50	27.8	27.5	16.7	
Relative concordance	33.5	50	39	50	39	39	
Discordance	11.2	16.6	11	22.2	33.5	44.3	
MRA versus GPS							
Concordance	55.3	55.4	66.2	55.3	44.5	39	66.5
Relative concordance	39.5	33.3	22.5	33.7	33.2	39	27.6
Discordance	5.2	11.3	11.3	11	22.3	22	5.9
iVue versus MRA							
Concordance	72.1	39	50	50.2	50	50	
Relative concordance	16.2	39	39	27.5	27.5	24	
Discordance	11.7	22	11	22.3	22.5	26	

**Table 3:** Inferotemporal (IT), inferonasal (IN), superotemporal (ST), superonasal (SN), nasal (N), and temporal (T) quadrants.

iVue versus GPS	Kappa test (SE)	Kappa test (SE)	Kappa test (SE)
	All patients	Normal VF	Abnormal VF
IT	0.225 (0.136)	0.059 (0.171)	0.555 (0.286)
IN	0.105 (0.134)	0.044 (0.150)	0.333 (0.304)
ST	0.404 (0.136)	0.225 (0.186)	0.173 (0.632)
SN	0.202 (0.133)	0.182 (0.152)	0.250 (0.279)
N	0.060 (0.125)	0.134 (0.152)	0.153 (0.232)
T	−0.015 (0.124)	−0.059 (0.161)	0.043 (0.176)

**Table 4:** Inferotemporal (IT), inferonasal (IN), superotemporal (ST), superonasal (SN), nasal (N), and temporal (T) quadrants.

GPS versus MRA	*κ*-test (SE)	*κ*-test (SE)	*κ*-test (SE)
	All patients	Normal VF	Abnormal VF
IT	0.618 (0.121)	0.516 (0.160)	0.792 (0.197)
IN	0.527 (0.123)	0.503 (0.142)	0.526 (0.295)
ST	0.448 (0.134)	0.314 (0.178)	0.560 (0.281)
SN	0.519 (0.131)	0.508 (0.161)	0.476 (0.257)
N	0.333 (0.139)	0.299 (0.169)	0.400 (0.244)
T	0.143 (0.154)	0.211 (0.203)	0.310 (0.267)
Global	0.511 (0.133)	0.391 (0.168)	0.782 (0.206)

**Table 5:** Inferotemporal (IT), inferonasal (IN), superotemporal (ST), superonasal (SN), nasal (N), and temporal (T) quadrants.

iVue versus MRA	Kappa test (SE)	Kappa test (SE)	Kappa test (SE)
	All patients	Normal VF	Abnormal VF
IT	0.585 (0.117)	0.465 (0.181)	0.545 (0.234)
IN	0.265 (0.165)	0.152 (0.270)	0.400 (0.244)
ST	0.602 (0.139)	0.370 (0.262)	0.607 (0.251)
SN	0.656 (0.132)	0.845 (0.106)	0.225 (0.316)
N	0.627 (0.142)	0.843 (0.107)	−0.200 (0.489)
T	−0.050 (0.405)	−0.023 (0.706)	−0.130 (0.470)
